# Comparison of Neoadjuvant TCHP and ddAC+THP Regimens for Pathologic Complete Response in HER2-Positive Breast Cancer: A Multicenter Real-World Analysis of Systemic Inflammatory Biomarkers

**DOI:** 10.3390/medicina62071370

**Published:** 2026-07-16

**Authors:** Gökhan Şahin, Ahmet Kürşad Dişli, Firat Sirvan, Mustafa Murat Mıdık, Nur Evsan Boyraz, Oben Belen, Taha Koray Şahin, Ayşe Nuransoy Cengiz, Fatih Kuş, Sıla Gökdere, Erdem Göker, Burcu Çakar, Sercan Aksoy, Mevlüde İnanç, Deniz Can Güven, Hasan Çağrı Yıldırım

**Affiliations:** 1 Department of Medical Oncology, Faculty of Medicine, Ege University, 35100 Izmir, Turkey; 2 Department of Medical Oncology, Faculty of Medicine, Erciyes University, 38039 Kayseri, Turkey; 3 Department of Internal Medicine, Faculty of Medicine, Hacettepe University, 06230 Ankara, Turkey; 4 Department of Internal Medicine, Faculty of Medicine, Ege University, 35100 Izmir, Turkey; 5 Department of Medical Oncology, Faculty of Medicine, Hacettepe University, 06230 Ankara, Turkey; 6 Department of Medical Oncology, Atatürk Sanatorium Training and Research Hospital, 06290 Ankara, Turkey

**Keywords:** HER2-positive breast cancer, neoadjuvant therapy, pathologic complete response, dual HER2 blockade, TCHP, ddAC+THP, systemic inflammation response index, SIRI, inflammatory biomarkers, real-world study

## Abstract

*Background and Objectives*: Neoadjuvant dual HER2 blockade combined with chemotherapy is the standard treatment approach for patients with high-risk early-stage or locally advanced HER2-positive breast cancer. However, the optimal chemotherapy backbone and the predictive value of systemic inflammatory biomarkers remain subjects of ongoing investigation. This study aimed to compare pathologic complete response (pCR) rates between neoadjuvant dose-dense doxorubicin/cyclophosphamide followed by paclitaxel plus trastuzumab and pertuzumab (ddAC+THP) and docetaxel, carboplatin, trastuzumab, and pertuzumab (TCHP), and to evaluate the predictive performance of pretreatment inflammatory biomarkers. *Materials and Methods***:** In this multicenter retrospective study, patients with HER2-positive breast cancer treated with neoadjuvant ddAC+THP or TCHP between 2019 and 2025 at three tertiary centers were evaluated. Pretreatment inflammatory biomarkers, including neutrophil-to-lymphocyte ratio (NLR), platelet-to-lymphocyte ratio (PLR), lymphocyte-to-monocyte ratio (LMR), systemic immune-inflammation index (SII), systemic inflammation response index (SIRI), and hemoglobin, albumin, lymphocyte, and platelet (HALP) score, were calculated from baseline laboratory parameters. Receiver operating characteristic analyses were performed to determine optimal cutoff values, and logistic regression analyses were used to identify predictors of pCR. *Results***:** A total of 197 patients were included, of whom 138 received ddAC+THP and 59 received TCHP. Overall, 125 patients (63.5%) achieved pCR. The pCR rate was numerically higher in the ddAC+THP group than in the TCHP group (65.2% vs. 59.3%), although the difference was not statistically significant (*p* = 0.431). Among the evaluated biomarkers, SIRI demonstrated the highest discriminatory performance for predicting pCR (AUC: 0.725, 95% CI: 0.652–0.797), followed by NLR (AUC: 0.673, 95% CI: 0.595–0.750). In multivariable analysis, hormone receptor positivity (OR: 0.291, 95% CI: 0.131–0.645; *p* = 0.002) and elevated SIRI (>0.845) (OR: 0.088, 95% CI: 0.036–0.216; *p* < 0.001) were independently associated with lower odds of achieving pCR. No significant difference in pCR was observed between treatment regimens across predefined subgroup analyses. *Conclusions***:** Neoadjuvant ddAC+THP and TCHP achieved comparable pCR outcomes in patients with HER2-positive breast cancer. SIRI was independently associated with a lower likelihood of achieving pCR and showed acceptable discriminatory performance. These findings suggest that SIRI may represent an exploratory, readily available inflammatory biomarker for pCR risk stratification; however, prospective validation is required before clinical application.

## 1. Introduction

Breast cancer remains the most frequently diagnosed malignancy and a leading cause of cancer-related mortality among women worldwide [1]. Human epidermal growth factor receptor 2 (HER2) overexpression or amplification is observed in approximately 15–20% of all breast cancer cases and is historically characterized by an aggressive clinical course, higher rates of recurrence, and poor overall survival [2]. However, the therapeutic landscape of HER2-positive (HER2+) breast cancer has been fundamentally transformed over the past few decades by the advent of anti-HER2 targeted therapies, moving from the monoclonal antibody trastuzumab to dual HER2 blockade combinations [3].

In contemporary clinical practice, neoadjuvant systemic therapy (NST) is widely established as the standard of care for patients with early-stage high-risk or locally advanced HER2+ breast cancer [4]. Neoadjuvant approach not only downstages tumor burden thereby increasing the feasibility of breast-conserving surgery and axillary downstaging but also serves as an invaluable in vivo dynamic assessment of therapeutic response [5]. Achieving a pathologic complete response (pCR), traditionally defined as the complete absence of residual invasive tumor in both the breast and axillary lymph nodes (ypT0/is ypN0) following NST, is widely recognized as a robust surrogate endpoint for favorable long-term oncological outcomes, including significantly prolonged disease-free survival (DFS) and overall survival (OS) [6]. To maximize pCR rates, modern neoadjuvant regimens frequently utilize dual HER2 inhibition by combining trastuzumab and pertuzumab with systemic chemotherapy. Two prominent treatment paradigms have emerged in this setting: anthracycline-containing regimens, such as dose-dense doxorubicin and cyclophosphamide followed by paclitaxel with dual HER2 blockade (ddAC+THP), and anthracycline-free regimens, most notably docetaxel, carboplatin, trastuzumab, and pertuzumab (TCHP) [7,8]. While anthracyclines have long been a cornerstone of breast cancer chemotherapy, concern regarding cumulative, potentially irreversible cardiotoxicity has led to increasing interest in non-anthracycline platinum-based regimens like TCHP [9]. Clinical trials and real-world evidence continue to debate whether the omission of anthracyclines compromises efficacy or optimizes the safety profile without sacrificing pCR rates, making the direct comparison of ddAC+THP and TCHP in clinical cohorts highly relevant [10,11]. In real-world practice, selection between these regimens is often influenced by patient characteristics, cardiac risk, institutional preference, and physician discretion, further supporting the need to evaluate their outcomes in routine clinical cohorts.

Beyond the choice of the chemotherapeutic backbone, there is a growing clinical imperative to identify reliable, minimally invasive, and cost-effective biomarkers capable of predicting pCR prior to the initiation of NST. Such biomarkers could facilitate personalized treatment escalation or de-escalation strategies. Emerging evidence highlights that the tumor microenvironment and systemic inflammatory responses play critical roles in breast cancer progression, angiogenesis, and resistance to systemic therapies [12]. Peripheral blood-based inflammatory indices derived from routine complete blood counts have gained substantial attention as surrogate markers of the host’s immune-inflammatory status. These include the neutrophil-to-lymphocyte ratio (NLR), platelet-to-lymphocyte ratio (PLR), lymphocyte-to-monocyte ratio (LMR), systemic immune-inflammation index (SII), and the systemic inflammation response index (SIRI) [13,14]. While elevated baseline inflammatory markers like NLR and SII have been associated with a lower likelihood of achieving pCR and poorer survival outcomes in various breast cancer subtypes, their comparative and independent predictive values specifically within HER2+ breast cancer patients undergoing dual HER2-targeted neoadjuvant regimens remain to be fully elucidated [15,16]. Furthermore, multi-center real-world data directly assessing the interplay between these systemic inflammatory biomarkers and specific chemotherapy backbones (anthracycline-based vs. anthracycline-free) are limited.

Therefore, the aim of this multicenter retrospective study was to compare the efficacy, in terms of pCR rates, between neoadjuvant ddAC+THP and TCHP regimens in patients with HER2+ breast cancer treated across three major tertiary centers. Additionally, we evaluated and compared the predictive performance of various pretreatment peripheral blood inflammatory biomarkers (NLR, PLR, LMR, SII, SIRI, and HALP) to identify independent predictors of pCR, aiming to contribute to more refined, individualized therapeutic strategies in this patient population.

## 2. Materials and Methods

### 2.1. Study Design and Patient Population

This multicenter retrospective study included patients diagnosed with HER2-positive breast cancer between 2019 and 2025 at Hacettepe University, Ankara, Türkiye; Ege University, İzmir, Türkiye; and Erciyes University, Kayseri, Türkiye, who were treated with neoadjuvant systemic therapy. After applying the exclusion criteria, 44 patients were excluded: 18 due to missing pretreatment laboratory parameters required for inflammatory biomarker calculation, 16 due to incomplete neoadjuvant treatment, and 10 due to unavailable postoperative pathologic response data. Finally, 197 patients were included in the final analysis.

Eligible patients were required to have histologically confirmed HER2-positive breast cancer and to have completed neoadjuvant HER2-targeted treatment followed by definitive breast surgery. HER2 status was assessed on pretreatment core biopsy specimens according to the American Society of Clinical Oncology/College of American Pathologists (ASCO/CAP) recommendations. HER2 positivity was defined as immunohistochemistry score 3+ or immunohistochemistry score 2+ with HER2 gene amplification confirmed by in situ hybridization. Patients with HER2-low disease, HER2 2+ without confirmed gene amplification, equivocal or unconfirmed HER2 status, or missing HER2 documentation were not included. HER2 status was verified from the original pathology reports to confirm eligibility according to ASCO/CAP-based criteria. Hormone receptor positivity was defined as estrogen receptor and/or progesterone receptor nuclear staining of ≥1% on immunohistochemistry.

Clinicopathological variables, including age, clinical T stage, clinical N stage, histologic subtype, lesion type, hormone receptor status, HER2 status, histologic grade, Ki-67 index, treatment regimen, and pathologic response data, were retrospectively collected from institutional electronic medical records. Patients with missing baseline laboratory parameters required for inflammatory biomarker calculations were excluded from the final analysis, and complete-case analysis was performed.

The study was approved by the Ege University Medical Research Ethics Committee on 21 August 2025 (approval number: 25-8T/46; protocol number: 2025-54O5).

### 2.2. Treatment Regimens and Definition of Pathologic Complete Response

Patients received one of the following neoadjuvant treatment regimens according to institutional preference and physician discretion:

- ddAC+THP: dose-dense doxorubicin and cyclophosphamide followed by paclitaxel combined with trastuzumab and pertuzumab;
- TCHP: docetaxel, carboplatin, trastuzumab, and pertuzumab.

Definitive breast surgery was performed following completion of neoadjuvant therapy. The primary endpoint of the study was pathologic complete response (pCR), defined as the absence of residual invasive disease in both the breast and axillary lymph nodes (ypT0/is, ypN0). Pathologic response data were extracted from routine postoperative pathology reports at each participating institution using the predefined pCR definition of ypT0/is, ypN0. A central pathology review of pCR status was not performed.

### 2.3. Inflammatory Biomarkers

Pretreatment peripheral blood parameters obtained within 2 weeks before initiation of neoadjuvant therapy were used to calculate inflammatory biomarkers. Blood samples were obtained before initiation of systemic therapy and before chemotherapy-related corticosteroid premedication. Data on active infection, chronic inflammatory conditions, or non-oncologic corticosteroid use at the time of blood sampling were not uniformly available across centers because of the retrospective design.

NLR was calculated as neutrophil count divided by lymphocyte count; LMR as lymphocyte count divided by monocyte count; PLR as platelet count divided by lymphocyte count; SII as platelet count × neutrophil count/lymphocyte count; SIRI as neutrophil count × monocyte count/lymphocyte count; and HALP as hemoglobin × albumin × lymphocyte count/platelet count.

Collinearity diagnostics among inflammatory biomarkers were evaluated using variance inflation factor (VIF) analysis. A VIF value < 5 was considered indicative of the absence of clinically significant multicollinearity. Receiver operating characteristic (ROC) analyses were subsequently performed to evaluate the discriminatory performance of inflammatory biomarkers for predicting pCR. Exploratory cutoff values were determined using the Youden index method. Biomarkers with relatively higher discriminatory performance (AUC > 0.60) were considered for subsequent exploratory predictive analyses.

### 2.4. Statistical Analysis

Continuous variables were summarized using median and interquartile range (IQR), whereas categorical variables were expressed as frequencies and percentages. Comparisons between treatment groups were performed using appropriate statistical tests according to variable distribution and type. Given the absence of a universally accepted cutoff value for Ki-67 in the neoadjuvant HER2-positive setting, Ki-67 was categorized into low and high groups based on the cohort median value (30%), consistent with previous retrospective biomarker studies. As a sensitivity analysis, Ki-67 was additionally evaluated as a continuous variable expressed per 10% increase. Univariate logistic regression analyses were conducted to identify factors associated with pCR. Treatment regimen was forced into the multivariable logistic regression model as the main exposure of interest. Additional variables were included if they showed statistical significance in univariate analysis (*p* < 0.05), including hormone receptor status, histologic grade, Ki-67, NLR, and SIRI, while the number of covariates was restricted to reduce the risk of model overfitting. Because treatment allocation was not randomized and the two treatment groups were unequal in size, treatment-regimen comparisons were interpreted cautiously.

Exploratory subgroup analyses were additionally performed to evaluate whether the association between treatment regimen and pCR differed across predefined clinicopathological and inflammatory biomarker subgroups. A two-sided *p* value < 0.05 was considered statistically significant. No formal adjustment for multiple comparisons was applied because the inflammatory biomarker analyses were exploratory and involved biologically correlated indices derived from overlapping blood cell parameters; therefore, these findings were interpreted as hypothesis-generating.

All statistical analyses were primarily performed using IBM SPSS Statistics for Windows, version 28.0.1.1 (IBM Corp., Armonk, NY, USA). ROC analyses, optimal cutoff determination using the Youden index method, and forest plot visualizations were additionally performed using R software within the RStudio environment (RStudio version 2024.12.1 + 563; Posit Software, PBC, Boston, MA, USA).

## 3. Results

### 3.1. Baseline Characteristics of Patients Treated with ddAC+THP or TCHP

Table 1 summarizes the baseline demographic, clinical, and pathological characteristics of the study cohort according to neoadjuvant treatment regimen. A total of 197 patients were included, of whom 138 (70.1%) received ddAC+THP and 59 (29.9%) received TCHP. The median age of the overall cohort was 49.0 years (IQR, 41.7–58.9), with no significant age difference between the treatment groups (*p* = 0.447). The majority of patients had cT1–2 tumors (83.8%) and cN2–3 nodal disease (51.3%). Clinical T stage and N stage distributions were similar between the ddAC+THP and TCHP groups (*p* = 0.276 and *p* = 0.938, respectively). Invasive ductal carcinoma was the predominant histologic subtype (78.7%), while 21.3% of patients had other histologies. Histologic subtype distribution did not significantly differ between treatment regimens (*p* = 0.358).

Single-lesion tumors were observed in 61.9% of patients, whereas 38.1% had multifocal or multicentric disease, with no significant intergroup difference (*p* = 0.153). Patients were categorized according to hormone receptor status as HER2-positive/hormone receptor-positive and HER2-positive/hormone receptor-negative disease. Overall, 125 patients (63.5%) had hormone receptor-positive disease and 72 patients (36.5%) had hormone receptor-negative disease. HER2 IHC 3+ expression was detected in 81.2% of patients. Hormone receptor status and HER2 status were balanced between the treatment groups (*p* = 0.614 and *p* = 0.667, respectively). Regarding tumor biology, 50.8% of patients had grade 3 tumors. The median Ki67 index was 30% (IQR, 20–50) in the overall cohort and did not significantly differ between treatment groups (*p* = 0.460). Similarly, the distribution of Ki67 groups (≤30% vs. >30%) was comparable between the ddAC+THP and TCHP groups (*p* = 0.962).

Overall, 125 patients (63.5%) achieved pathologic complete response (pCR). Although the pCR rate was numerically higher in the ddAC+THP group compared with the TCHP group (65.2% vs. 59.3%), the difference was not statistically significant (*p* = 0.431).

### 3.2. Predictive Performance of Inflammatory Biomarkers for pCR

To evaluate potential multicollinearity among inflammatory biomarkers, collinearity diagnostics were performed using variance inflation factor (VIF) analysis (Supplementary Table S1). No evidence of severe multicollinearity was observed, as all VIF values were below 5. Among the evaluated biomarkers, SII demonstrated the highest VIF value (4.933), followed by PLR (3.310) and NLR (3.014), whereas LMR, HALP, and SIRI showed lower collinearity indices. Therefore, all biomarkers were retained for subsequent ROC analyses.

**Figure 1:**
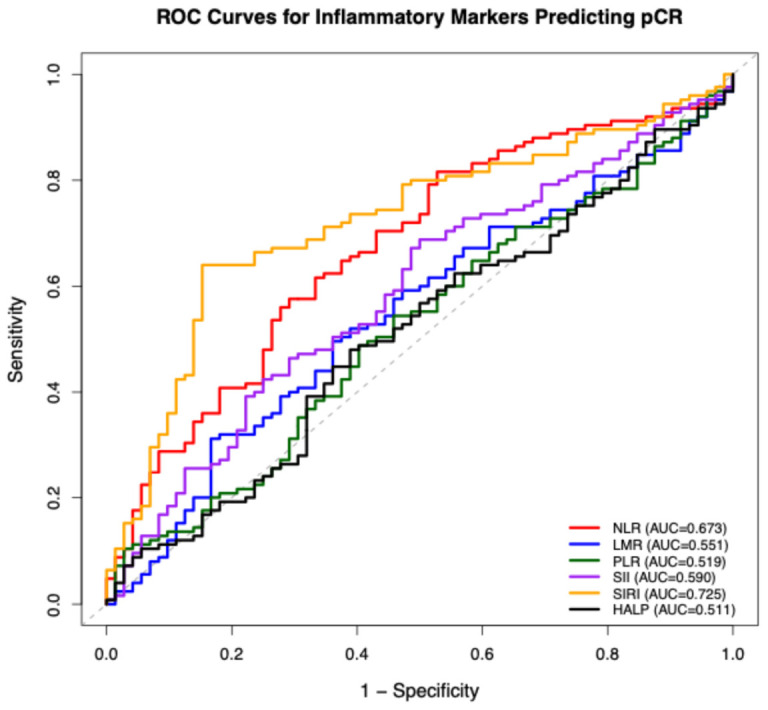
ROC curves of inflammatory biomarkers for predicting pCR.

The discriminatory performances of inflammatory biomarkers for predicting pCR are summarized in Table 2 and illustrated in Figure 1. Among the evaluated biomarkers, SIRI demonstrated the highest discriminatory predictive performance, with an AUC of 0.725 (95% CI, 0.652–0.797), corresponding to acceptable discrimination, followed by NLR with an AUC of 0.673 (95% CI, 0.595–0.750). SIRI yielded an optimal cutoff value of 0.845, corresponding to a sensitivity of 64.0% and specificity of 84.7%, whereas NLR showed a higher sensitivity (81.6%) but lower specificity (47.2%) at a cutoff value of 2.74.

SII demonstrated modest discriminatory ability, with an AUC of 0.590 (95% CI, 0.509–0.672), while LMR, PLR, and HALP showed limited predictive performance, with AUC values close to 0.50. Based on the predefined criterion of AUC > 0.60 for acceptable discriminatory performance, only SIRI and NLR were selected for subsequent predictive analyses evaluating factors associated with pCR.

### 3.3. Univariate Logistic Regression Analysis for Predictors of pCR

Univariate logistic regression analysis for predictors of pCR is presented in Table 3. No significant association was observed between neoadjuvant treatment regimen and pCR achievement, as the odds of achieving pCR were comparable between patients treated with TCHP and those receiving ddAC+THP (OR, 0.778; 95% CI, 0.416–1.455; *p* = 0.432). Similarly, age group, clinical T stage, clinical N stage, lesion type, and HER2 status were not significantly associated with pCR (all *p* > 0.05). In contrast, several clinicopathological factors demonstrated significant associations with pCR. Compared with invasive ductal carcinoma, other histologic subtypes were associated with lower odds of achieving pCR (OR, 0.433; 95% CI, 0.217–0.866; *p* = 0.018). HR-positive tumors also showed significantly lower pCR rates compared with HR-negative tumors (OR, 0.314; 95% CI, 0.161–0.613; *p* < 0.001).

Higher histologic grade and elevated Ki-67 status were significantly associated with increased likelihood of pCR. Grade 3 tumors were associated with higher odds of pCR compared with grade 2 tumors (OR, 1.950; 95% CI, 1.082–3.514; *p* = 0.026), while high Ki-67 status was associated with a greater probability of pCR relative to low Ki-67 status (OR, 2.111; 95% CI, 1.163–3.830; *p* = 0.014). When Ki-67 was evaluated as a continuous variable, each 10% increase in Ki-67 was also associated with higher odds of pCR (OR, 1.46; 95% CI, 1.20–1.79; *p* < 0.001).

Among inflammatory biomarkers, both NLR and SIRI demonstrated significant associations with pCR. High NLR (≥2.74) was associated with lower odds of achieving pCR compared with low NLR (<2.74) (OR, 0.252; 95% CI, 0.132–0.481; *p* < 0.001). Likewise, high SIRI (≥0.845) was strongly associated with reduced pCR likelihood (OR, 0.101; 95% CI, 0.048–0.212; *p* < 0.001).

### 3.4. Multivariate Logistic Regression Analysis for Predictors of pCR

Variables demonstrating clinical relevance and/or significant associations in univariate analyses were included in the multivariate logistic regression model for predictors of pCR (Table 4). After adjustment for potential confounding factors, treatment regimen was not independently associated with pCR (TCHP vs. ddAC+THP: OR, 1.93; 95% CI, 0.82–4.57; *p* = 0.133). HR positivity remained independently associated with lower odds of achieving pCR compared with HR-negative disease (OR, 0.27; 95% CI, 0.12–0.62; *p* = 0.002). When Ki-67 was evaluated as a continuous variable, each 10% increase in Ki-67 was independently associated with higher odds of pCR (OR, 1.35; 95% CI, 1.07–1.70; *p* = 0.010). Histologic grade and NLR were not independently associated with pCR after multivariate adjustment. High SIRI remained independently associated with reduced pCR likelihood (OR, 0.07; 95% CI, 0.03–0.19; *p* < 0.001).

Overall, SIRI remained the only inflammatory biomarker independently associated with lower pCR likelihood in the adjusted model.

### 3.5. Subgroup Analysis According to Neoadjuvant Treatment Regimen

**Figure 2:**
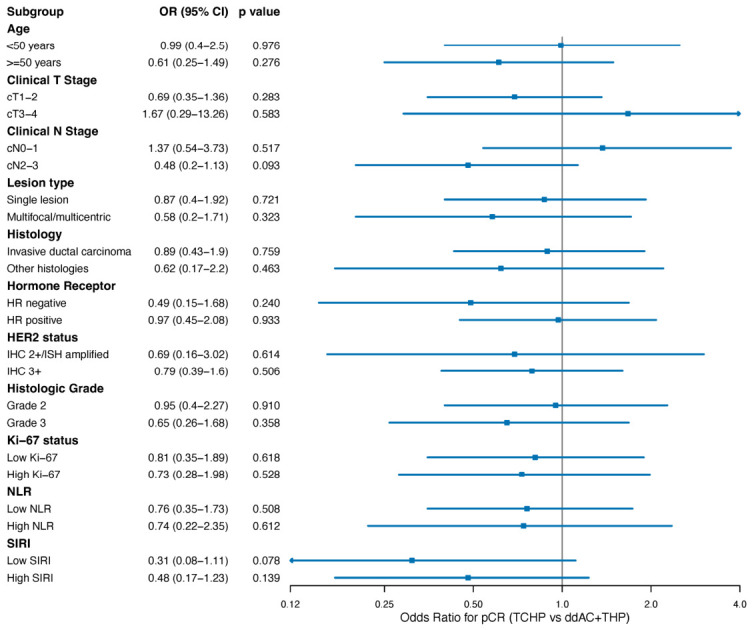
Exploratory subgroup analysis of pCR according to neoadjuvant treatment regimen (TCHP vs. ddAC+THP).

Exploratory subgroup analyses were performed to evaluate whether the association between neoadjuvant treatment regimen and pCR differed across clinicopathological and inflammatory biomarker subgroups (Figure 2). Across all evaluated subgroups, no statistically significant difference in pCR odds was observed between patients treated with TCHP and those receiving ddAC+THP, as all confidence intervals crossed the null value.

Exploratory subgroup analyses did not identify a statistically significant treatment-regimen advantage for either TCHP or ddAC+THP across the evaluated clinicopathological and inflammatory biomarker subgroups. Although numerically lower odds of pCR with TCHP were observed in certain subgroups, including patients with cN2–3 disease and low SIRI levels, these differences did not reach statistical significance.

## 4. Discussion

In this multicenter retrospective study, we evaluated the clinical efficacy of anthracycline-containing (ddAC+THP) versus anthracycline-free (TCHP) neoadjuvant regimens in patients with HER2+ breast cancer and investigated the predictive capacity of comprehensive pretreatment inflammatory biomarkers for pCR. Our findings demonstrate that both regimens yielded high pCR rates, with no statistically significant difference observed between ddAC+THP and TCHP. Most notably, the Systemic Inflammation Response Index (SIRI) remained independently associated with a lower likelihood of achieving pCR and showed stronger statistical performance than traditional markers such as the Neutrophil-to-Lymphocyte Ratio (NLR) in multivariate analysis.

The optimal chemotherapy backbone combined with dual HER2 blockade in the neoadjuvant setting remains a subject of intense clinical debate. Historically, anthracyclines have been considered indispensable components of breast cancer regimens. However, their use is notoriously constrained by cumulative, irreversible cardiotoxicity and an elevated risk of secondary malignancies [17,18]. The landmark TRAIN-2 study directly addressed this concern, demonstrating that the omission of anthracyclines from a neoadjuvant regimen containing dual HER2 blockade did not compromise pCR rates or three-year disease-free survival, while significantly mitigating cardiotoxicity [8,19]. Similarly, the cardiac safety and high efficacy of the anthracycline-free TCHP regimen were underscored in the TRYPHAENA trial [7]. In alignment with these prospective data, our real-world cohort showed that the pCR rate was numerically higher in the ddAC+THP group compared to the TCHP group (65.2% vs. 59.3%), but this difference did not reach statistical significance (*p* = 0.431). Exploratory subgroup analyses did not suggest a consistent statistically significant advantage for either treatment backbone across distinct clinicopathological or inflammatory risk strata; however, these findings should be interpreted cautiously because of limited subgroup sample sizes. These findings further support the growing clinical use of anthracycline-free regimens such as TCHP as a feasible therapeutic alternative in appropriately selected patients.

Beyond the chemotherapy backbone, identifying biomarkers that reflect the biological heterogeneity of HER2+ tumors and the host immune environment is crucial for refining personalized medicine. In recent years, the intricate interplay between systemic inflammation, the tumor microenvironment, and treatment resistance has been widely acknowledged [20]. Peripheral immune cells, including neutrophils, lymphocytes, platelets, and monocytes, play distinct roles in this cascade. Neutrophils can promote tumor growth, angiogenesis, and suppress T-cell-mediated antitumor immunity via the secretion of circulating cytokines and vascular endothelial growth factors [21]. Conversely, tumor-infiltrating lymphocytes (TILs) are vital for executing cell-mediated cytotoxicity and mediating the therapeutic effects of anti-HER2 monoclonal antibodies through antibody-dependent cellular cytotoxicity (ADCC) [22,23]. Consequently, peripheral indices derived from these cells have been extensively studied.

In our study, univariate analysis indicated that elevated baseline NLR (≥2.74) and SIRI (≥0.845) were robustly associated with reduced odds of achieving pCR (*p* < 0.001). These findings are consistent with several previous reports suggesting that high baseline systemic inflammation may reflect a less favorable host immune context associated with reduced response to neoadjuvant systemic therapies across various breast cancer subtypes [24,25]. However, when these markers were subjected to multivariate logistic regression to adjust for potential confounding clinical variables, NLR lost its statistical significance, whereas SIRI remained a statistically significant and independent predictor of poor pathologic response.

This divergent behavior between NLR and SIRI represents a key finding of our study. While NLR captures the balance between neutrophils and lymphocytes, SIRI integrates three distinct leukocyte subsets: neutrophils, monocytes, and lymphocytes (SIRI = neutrophils × monocytes/lymphocytes). Monocytes may contribute to tumor-promoting inflammation and can serve as precursors of tumor-associated macrophages, which have been implicated in immunosuppressive tumor microenvironments [26]. However, because our study did not include tumor immune profiling, macrophage markers, cytokine measurements, TIL quantification, or HER2 immune-effector correlates, these mechanistic links remain speculative. Therefore, SIRI should be interpreted as a readily available systemic inflammatory biomarker rather than a direct surrogate of the tumor immune microenvironment. By incorporating neutrophil, monocyte, and lymphocyte components, SIRI may provide a broader systemic inflammatory signal than NLR or PLR alone. This may explain why SIRI exhibited the highest discriminatory performance among the evaluated biomarkers in our ROC analysis (AUC 0.725), corresponding to acceptable discrimination, and retained independent predictive power in multivariate models. Our results mirror findings in other aggressive malignancies where SIRI outperformed conventional dual-cell ratios as a prognostic and predictive tool [27,28]. Nevertheless, despite being the best-performing inflammatory biomarker in our analysis, the overall discriminatory capacity of SIRI remained moderate, suggesting that inflammatory biomarkers alone are unlikely to fully capture the biological complexity underlying treatment response. This cautious interpretation is also supported by previous neoadjuvant HER2-positive breast cancer studies reporting inconsistent or limited predictive relevance for SIRI and related inflammatory biomarkers, including NLR, PLR, LMR, SII, and monocyte-related indices, particularly when evaluated as stand-alone markers [29,30]. Therefore, SIRI should not be used as a stand-alone tool for clinical decision-making or treatment selection. Rather, it may provide complementary, hypothesis-generating information when interpreted together with established clinicopathological factors such as hormone receptor status, tumor stage, histologic grade, and Ki-67, pending prospective validation in integrated predictive models.

Consistent with established literature, our multivariate analysis also confirmed that hormone receptor (HR) positivity was independently associated with a lower probability of achieving pCR (OR 0.291, *p* = 0.002). It is well-documented that HER2+/HR+ tumors exhibit distinct biological behaviors compared to HER2+/HR- tumors, frequently demonstrating lower sensitivity to standard neoadjuvant chemotherapy backbones due to cross-talk between the estrogen receptor and HER2 signaling pathways [31,32]. Interestingly, while high histologic grade and high Ki-67 index (≥30%) were significantly correlated with higher pCR rates in univariate analysis [33], only Ki-67 remained independently associated with pCR when evaluated as a continuous variable in the multivariable model. In contrast, histologic grade lost statistical significance after multivariable adjustment, whereas SIRI remained independently associated with lower pCR likelihood, supporting its potential role as a hypothesis-generating systemic inflammatory biomarker.

In addition to systemic inflammatory biomarkers, tissue-based pathological features such as tumor-infiltrating lymphocytes and lymphovascular invasion have also been associated with neoadjuvant treatment response in HER2-positive breast cancer [34,35]. These parameters may provide complementary information regarding the local immune microenvironment and tumor invasiveness. However, in the present retrospective multicenter cohort, standardized data on tumor-infiltrating lymphocytes and lymphovascular invasion were not uniformly available across centers, precluding their reliable incorporation into the analysis.

Several limitations of this study should be acknowledged. First, the retrospective design introduces an inherent risk of selection bias and unmeasured confounding. Treatment allocation was not randomized and was influenced by institutional practices and physician discretion, which may have contributed to imbalance between treatment groups. The unequal group sizes, particularly the smaller number of patients in the TCHP group, limited the statistical power of treatment-regimen comparisons. Because propensity score matching or inverse probability weighting was not performed and detailed treatment-delivery or clinical management variables, including body mass index, comorbidities, performance status, relative dose intensity, treatment delays, toxicity, cardiac events, radiologic response, surgery type, and adjuvant escalation strategies, were not uniformly available across centers, the treatment-regimen comparison should be interpreted as an exploratory pCR-based analysis rather than a comprehensive efficacy-safety comparison. Second, the exclusion of patients with missing laboratory parameters may have introduced additional selection bias. Third, although SIRI demonstrated independent predictive significance, the overall discriminatory performance of inflammatory biomarkers remained moderate, and ROC-derived cutoffs were not internally or externally validated; therefore, these findings should be considered hypothesis-generating. Furthermore, survival outcomes such as disease-free survival and overall survival were not evaluated, limiting long-term clinical interpretation. Serial inflammatory parameters after neoadjuvant therapy and surgery were also not uniformly available, precluding assessment of longitudinal treatment-related changes in these biomarkers.

Additionally, a full central pathological re-evaluation of all archival tumor specimens was not performed because of the retrospective multicenter design. Nevertheless, HER2 positivity was verified from the original pathology reports according to ASCO/CAP-based eligibility criteria. Standardized data on lymphovascular invasion and tumor-infiltrating lymphocytes were not consistently available across centers; therefore, these potentially relevant pathological biomarkers could not be incorporated into the analysis.

## 5. Conclusions

In conclusion, our multicenter real-world analysis demonstrated that anthracycline-containing and anthracycline-free neoadjuvant dual HER2 blockade regimens were associated with high pCR rates, with no statistically significant difference observed between groups. Among evaluated inflammatory biomarkers, SIRI emerged as the most informative independent inflammatory predictor of a lower likelihood of achieving pCR, potentially reflecting the combined influence of systemic inflammation and immunosuppressive tumor microenvironment dynamics. These findings suggest that inflammation-based biomarkers may warrant further investigation for pCR risk stratification, while highlighting the need for prospective validation in larger cohorts.

## Figures and Tables

**Table 1:** Baseline demographic and clinical characteristics according to neoadjuvant treatment regimen.

Characteristics	All Patients (N = 197)	ddAC+THP (N = 138)	TCHP (N = 59)	*p* Value
**Age, years, median (IQR)**	49.0 (41.7–58.9)	48.6 (41.6–57.7)	51.0 (41.7–59.4)	0.447
**Clinical T stage**				0.276
cT1–2	165 (83.8%)	113 (81.9%)	52 (88.1%)	
cT3–4	32 (16.2%)	25 (18.1%)	7 (11.9%)	
**Clinical N stage**				0.938
cN0–1	96 (48.7%)	67 (48.6%)	29 (49.2%)	
cN2–3	101 (51.3%)	71 (51.4%)	30 (50.8%)	
**Histology**				0.358
Invasive ductal carcinoma	155 (78.7%)	111 (80.4%)	44 (74.6%)	
Other histologies *	42 (21.3%)	27 (19.6%)	15 (25.4%)	
**Lesion type**				0.153
Single lesion	122 (61.9%)	81 (58.7%)	41 (69.5%)	
Multifocal/multicentric	75 (38.1%)	57 (41.3%)	18 (30.5%)	
**Hormone receptor status**				0.614
HR negative	72 (36.5%)	52 (37.7%)	20 (33.9%)	
HR positive	125 (63.5%)	86 (62.3%)	39 (66.1%)	
**HER2 status**				0.667
IHC 2+/ISH amplified	37 (18.8%)	27 (19.6%)	10 (16.9%)	
IHC 3+	160 (81.2%)	111 (80.4%)	49 (83.1%)	
**Histologic grade**				0.544
Grade 2	97 (49.2%)	66 (47.8%)	31 (52.5%)	
Grade 3	100 (50.8%)	72 (52.2%)	28 (47.5%)	
**Ki67, %, median (IQR)**	30 (20–50)	30 (25–50)	30 (22.5–40)	0.460
**Ki-67 group**				0.962
Ki-67 ≤ 30%	103 (52.3%)	72 (52.2%)	31 (52.5%)	
Ki-67 > 30%	94 (47.7%)	66 (47.8%)	28 (47.5%)	
**Pathologic complete response**				0.431
pCR	125 (63.5%)	90 (65.2%)	35 (59.3%)	
Non-pCR	72 (36.5%)	48 (34.8%)	24 (40.7%)	

* Including invasive lobular carcinoma, NOS carcinoma, apocrine carcinoma, micropapillary carcinoma, and other rare histologies. Abbreviations: HR, hormone receptor; ISH, in situ hybridization; pCR, pathologic complete response. Definitions: Hormone receptor positivity was defined as ER and/or PR positivity. pCR was defined as ypT0/is ypN0.

**Table 2:** ROC analysis of inflammatory biomarkers for predicting pCR.

Marker	AUC (95% CI)	Optimal Cutoff	Sensitivity	Specificity
NLR	0.673 (0.595–0.750)	2.74	81.6%	47.2%
LMR	0.551 (0.469–0.634)	5.05	31.2%	83.3%
PLR	0.519 (0.436–0.602)	138.8	54.4%	54.2%
SII	0.590 (0.509–0.672)	632.5	68.8%	50.0%
SIRI	0.725 (0.652–0.797)	0.845	64.0%	84.7%
HALP	0.511 (0.428–0.595)	43.98	48.0%	61.1%

**Abbreviations:** AUC, area under the curve; CI, confidence interval; HALP, hemoglobin–albumin–lymphocyte–platelet score; LMR, lymphocyte-to-monocyte ratio; NLR, neutrophil-to-lymphocyte ratio; pCR, pathologic complete response; PLR, platelet-to-lymphocyte ratio; SII, systemic immune-inflammation index; SIRI, systemic inflammation response index. **Footnote:** ROC analyses were performed using the DeLong method for estimation of 95% confidence intervals.

**Table 3:** Univariate logistic regression analysis for predictors of pCR.

Variable	Comparison	OR for pCR	95% CI	*p* Value
Treatment regimen	TCHP vs. ddAC+THP	0.778	0.416–1.455	0.432
Age group	≥50 years vs. <50 years	1.023	0.572–1.831	0.939
Clinical T stage	cT3–4 vs. cT1–2	0.952	0.435–2.083	0.903
Clinical N stage	cN2–3 vs. cN0–1	0.698	0.389–1.251	0.227
Histology	Other histologies vs. invasive ductal carcinoma	0.433	0.217–0.866	**0.018**
Lesion type	Multifocal/multicentric vs. single lesion	0.788	0.435–1.426	0.431
Hormone receptor status	HR positive vs. HR negative	0.314	0.161–0.613	**<0.001**
HER2 status	IHC 3+ vs. IHC2+/ISH amplified	1.415	0.684–2.927	0.349
Histologic grade	Grade 3 vs. Grade 2	1.950	1.082–3.514	**0.026**
Ki-67 status	High Ki-67 vs. low Ki-67	2.111	1.163–3.830	**0.014**
Ki-67 continuous	Per 10% increase	1.46	1.20–1.79	**<0.001**
NLR	High NLR (≥2.74) vs. low NLR (<2.74)	0.252	0.132–0.481	**<0.001**
SIRI	High SIRI (≥0.845) vs. low SIRI (<0.845)	0.101	0.048–0.212	**<0.001**

Abbreviations: CI, confidence interval; HER2, human epidermal growth factor receptor 2; HR, hormone receptor; IHC, immunohistochemistry; ISH, in situ hybridization; NLR, neutrophil-to-lymphocyte ratio; OR, odds ratio; pCR, pathologic complete response; SIRI, systemic inflammation response index. Bold values indicate statistically significant results (*p* < 0.05).

**Table 4:** Multivariate logistic regression analysis for predictors of pathologic complete response (pCR).

Variable	Comparison	OR for pCR	95% CI	*p* Value
Treatment regimen	TCHP vs. ddAC+THP	1.93	0.82–4.57	0.133
Hormone receptor status	HR positive vs. HR negative	0.27	0.12–0.62	**0.002**
Histologic grade	Grade 3 vs. Grade 2	1.15	0.53–2.52	0.727
Ki-67	Per 10% increase	1.35	1.07–1.70	**0.010**
NLR	High NLR (≥2.74) vs. low NLR (<2.74)	1.15	0.48–2.75	0.750
SIRI	High SIRI (≥0.845) vs. low SIRI (<0.845)	0.07	0.03–0.19	**<0.001**

**Abbreviations:** CI, confidence interval; HR, hormone receptor; NLR, neutrophil-to-lymphocyte ratio; OR, odds ratio; pCR, pathologic complete response; SIRI, systemic inflammation response index. Bold values indicate statistically significant results (*p* < 0.05).

## Data Availability

The data presented in this study are available on request from the corresponding author.

## Supplementary Materials

The following supporting information can be downloaded at: https://www.mdpi.com/article/10.3390/medicina62071370/s1, Table S1. Collinearity diagnostics among inflammatory biomarkers.
