# Supplementary material for: Comparison of Neoadjuvant TCHP and ddAC+THP Regimens for Pathologic Complete Response in HER2-Positive Breast Cancer: A Multicenter Real-World Analysis of Systemic Inflammatory Biomarkers
Source: Medicina (Kaunas). 2026 Jul 16;62(7):1370. doi: 10.3390/medicina62071370 (PMC13414168; doi:10.3390/medicina62071370)
Supplement: Supplementary file 1 [file medicina-62-01370-s001.zip › medicina-4396545-supplementary.pdf]

Supplementary Table S1. Collinearity diagnostics among inflammatory biomarkers

Variable	Tolerance	VIF
NLR	0.332	3.014
LMR	0.733	1.364
PLR	0.302	3.310
SII	0.203	4.933
HALP	0.676	1.479
SIRI	0.515	1.942

Abbreviations: HALP, hemoglobin-albumin-lymphocyte-platelet score; LMR, lymphocyte-to-monocyte ratio; NLR, neutrophil-to-lymphocyte ratio; PLR, platelet-to-lymphocyte ratio; SII, systemic immune-inflammation index; SIRI, systemic inflammation response index; VIF, variance inflation factor.

Footnote: No severe multicollinearity was detected among inflammatory biomarkers, as all variance inflation factor (VIF) values were below 5.
